# Progerin impairs chromosome maintenance by depleting CENP-F from metaphase kinetochores in Hutchinson-Gilford progeria fibroblasts

**DOI:** 10.18632/oncotarget.8267

**Published:** 2016-03-22

**Authors:** Veronika Eisch, Xiang Lu, Diana Gabriel, Karima Djabali

**Affiliations:** ^1^ Epigenetics of Aging, Department of Dermatology, TUM School of Medicine, Technical University Munich (TUM), Garching-Munich, Germany

**Keywords:** progerin, lamin, mitosis, CENP-F, Hutchinson-Gilford progeria

## Abstract

Hutchinson-Gilford progeria syndrome (HGPS, OMIM 176670) is a rare premature aging disorder that leads to death at an average age of 14.7 years due to myocardial infarction or stroke. The most common mutation in HGPS is at position G608G (GGC>GGT) within exon 11 of the *LMNA* gene. This mutation results in the deletion of 50 amino acids at the carboxyl-terminal tail of prelamin A, producing a truncated farnesylated protein called progerin. Lamins play important roles in the organization and structure of the nucleus. The nuclear build-up of progerin causes severe morphological and functional changes in interphase HGPS cells. In this study, we investigated whether progerin elicits spatiotemporal deviations in mitotic processes in HGPS fibroblasts. We analyzed the nuclear distribution of endogenous progerin during mitosis in relation to components of the nuclear lamina, nuclear envelope (NE) and nuclear pores. We found that progerin caused defects in chromosome segregation as early as metaphase, delayed NE reformation and trapped lamina components and inner NE proteins in the endoplasmic reticulum at the end of mitosis. Progerin displaced the centromere protein F (CENP-F) from metaphase chromosome kinetochores, which caused increased chromatin lagging, binucleated cells and genomic instability. This accumulation of progerin-dependent defects with each round of mitosis predisposes cells to premature senescence.

## INTRODUCTION

Hutchinson-Gilford progeria syndrome (HGPS) is a rare, sporadic genetic disorder with phenotypic features of premature aging [1]. HGPS is caused by *de novo* dominant mutations in *LMNA* [2, 3]. *LMNA* encodes A-type nuclear lamins, and the predominant somatic cell isoforms, lamin A and lamin C arise by alternative RNA splicing [4]. Lamins are intermediate filament proteins that polymerize to form the nuclear lamina, a meshwork associated with the inner nuclear membrane (INM).

Lamin A is synthesized as a precursor, prelamin A, which has a CaaX motif at its carboxyl-terminus. The CaaX motif signals a series of catalytic reactions that result in the farnesylation and carboxymethylation of a carboxyl-terminal cysteine [5]. Farnesylated, carboxymethylated prelamin A is normally cleaved near its carboxyl-terminus by the ZMPSTE24 endoprotease, that results in the removal of the farnesylated cysteine [5]. The *LMNA* G608G mutation, which is responsible for the majority of cases of HGPS, creates an abnormal splice donor site within exon 11, generating an mRNA that encodes a prelamin A with a 50 amino acid deletion at its carboxyl-terminal domain [4]. Because the ZMPSTE24 endoproteolytic site is deleted in progerin carboxyl-terminus, this abnormal protein remains farnesylated [6]. The expression of progerin induces severe abnormalities in nuclear morphology, heterochromatin organization, mitosis, DNA replication and DNA repair [7-10]. Several *in vitro* and *in vivo* studies have now established that blocking the farnesylation step by using farnesyltransferase inhibitors (FTIs) reverses abnormalities in nuclear morphology in progerin-expressing cells [4, 11-18]. These studies have clearly implicated farnesylated progerin in HGPS pathogenesis, but the precise molecular mechanisms that govern how farnesylated progerin induces HGPS pathology remain to be investigated. Initial studies of progerin localization during mitosis in HeLa-transfected cells have provided the following major findings: (1) the anchorage of progerin to the INM disrupts the normal nuclear envelope (NE) disassembly, leading to the accumulation of progerin-membrane aggregates during mitosis [19, 20]; (2) these cytoplasmic progerin aggregates are associated with the INM protein SUN1 [21]; and (3) progerin's farnesyl moiety is partially responsible for these mitotic defects because blocking this protein modification using FTIs ameliorated these alterations [19-21]. These previous studies used HeLa cell models and therefore could not analyze the dynamics of endogenous progerin distribution in HGPS patient cells. Therefore, we investigated progerin repartitioning in mitotic HGPS fibroblasts in relation to normal cells. From the comparison between progerin distribution and components from the NE, nuclear pores, nuclear lamina, kinetochores, chromosomes, microtubules and endoplasmic reticulum, we discuss progerin-dependent mechanisms that may lead to increased levels of lagging chromatin, binucleated cells and genomic instability.

## RESULTS

To determine how progerin elicits phenotypic changes in HGPS fibroblasts during interphase, we investigated the spatiotemporal distribution of progerin during the different stages of mitosis in relation to distributions of the following proteins: A- and B-type lamins, the integral inner NE proteins emerin and SUN1, nuclear pores, the endoplasmic reticulum (ER) marker calnexin, microtubules, DNA, and the centromeric proteins CENP-E and CENP-F. We tracked the temporal sequence of progerin distribution patterns in 3 normal and 3 HGPS fibroblast lines using a specific anti-progerin antibody [22]. Fibroblast cultures were used between passages 10 to 16 and exhibited an average mitotic index of approximately 1.41% in the control and 0.95% in the HGPS cultures.

### Progerin, lamin A/C and lamin B1 dynamics in mitotic HGPS cells

**Figure 1:**
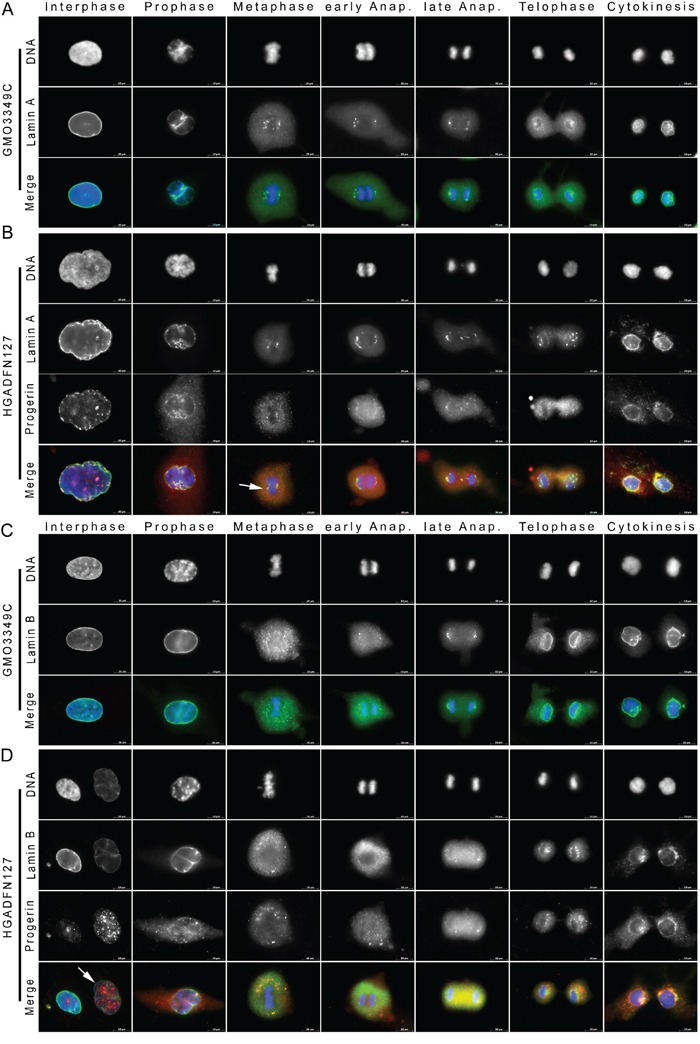
Cytoplasmic lamin A and lamin B1 aggregates colocalize with progerin during late cytokinesis in HGPS fibroblasts Immunocytochemistry was performed on fibroblasts from **A.** unaffected control (GMO3349C) and **B.** a subject with HGPS (HGADFN127). Cells were stained with anti-progerin antibody [22] and anti-lamin A antibody. Chromatin was stained with DAPI (blue). Representative staining of lamin A and progerin during the different stages of mitosis are shown as indicated (n=71). Single immunofluorescence images are shown in black and white. Progerin was not detected in mitotic control cells, therefore these single fluorescence images are not shown. The triple merged signals correspond to lamin A (green), progerin (red), and DAPI (blue) as indicated. In HGPS cells, progerin colocalized with lamin A foci at the core region of metaphase chromosomes (arrowhead), and in contrast to the control cells, lamin A and progerin remained mostly cytoplasmic during telophase. **C.** Immunofluorescence staining of control (GMO3349C) and **D.** HGPS (HGADFN127) fibroblasts using anti-progerin and anti-lamin B1 antibodies are shown as single images (n=44). The triple merged signals for lamin B1 (green), progerin (red) and chromatin (blue) are shown. The arrowhead indicates an HGPS nucleus with bright-progerin and low-lamin B1 signals. Cytoplasmic lamin B1 aggregates colocalized with progerin during cytokinesis. Scale bar, 10 μm.

In normal fibroblasts (Figure 1A), during NE breakdown in prophase, lamin A was delocalized from the disassembling nuclear lamina, which was detected as a thin discontinuous nuclear rim with anti-lamin A antibodies (Figure 1A). During metaphase, lamin A diffused throughout the cytoplasm and formed a few brightly labeled, distinct structures that were linearly distributed to the spindle pole side and core region of metaphase chromosomes (Figure 1A). In anaphase, lamin A was recruited to migrating chromosomes. During telophase, lamin A surrounded the newly formed nuclei and displayed a thin, irregular rim staining. During cytokinesis, a typical lamin A rim pattern was detected (Figure 1A). Progerin was not detected in the normal mitotic fibroblasts.

Interphase HGPS cells exhibit dysmorphic nuclei with NE invaginations and blebs [7, 8]. Progerin localized to the NE periphery and showed thickening of the lamina in some regions. Progerin aggregates were distributed throughout the nucleus (Figure 1B). Progerin signal was not homogenous, some nuclei were brightly labeled, whereas others were dim, indicating that progerin levels were variable [23]. HGPS mitotic cells also exhibited difference in signal intensity for progerin, with approximately 50% harboring a weak or strong signal, as shown in the statistic evaluation (Figure S1). During prophase, progerin appeared granular in the cytoplasm and some remained at the chromatin periphery (Figure 1B). During metaphase, progerin was distributed throughout the cytoplasm as aggregated structures, with a few of them localized to the core region of the metaphase chromosomes. The distinct progerin deposits at the spindle pole side of the chromosomes were superimposable with the ones labeled for lamin A (Figure 1B). Lamin A, however, remained diffused in the cytoplasm of HGPS cells during anaphase. In late anaphase, when lamin A began to surround migrating chromosomes, progerin retained a granular distribution in the cytoplasm (Figure 1B). During telophase, lamin A was not fully recruited to the chromosomes periphery. During cytokinesis, lamin A rim staining was detected, with some lamin A aggregates persisting in the cytoplasm (Figure 1B). During the same stage, progerin showed similar but thinner rim staining, and large amount of progerin remained in the cytoplasm (Figure 1B). Collectively, these data indicate that in HGPS cells, the presence of progerin disrupted the normal localization of lamin A to the spindle pole side of metaphase chromosomes, which appeared less confined. Progerin also delayed the recruitment of lamin A to the chromosomes during anaphase and telophase. Consequently, a small amount of cytoplasmic lamin A remained until late cytokinesis (Figure 1B). Progerin recruitment to the nuclear compartment was even more disrupted, as indicated by the large amounts of progerin aggregates that remained in the cytoplasm during cytokinesis. Progerin distribution was largely distinct from that of lamin A, and colocalization was observed, only at sites where lamin A formed aggregates (Figure 1B). Overall, lamin A was mislocalized in approximately 25% of the mitotic HGPS cells with a weak progerin signal and 96% of the cells that exhibited a strong progerin signal (Figure S1B).

We next investigated the distribution of lamin C in relation to lamin A (Figure S2). In normal cells, lamin C and lamin A showed similar distribution patterns, with a few aggregates linearly distributed to the core region of metaphase chromosomes until early anaphase (Figure S2A). By telophase, lamin C surrounded the surface of the segregated chromosomes, as previously reported [24] (Figure S2A).

In HGPS cells, lamin A and lamin C exhibited similar patterns and temporal distributions during mitosis, indicating that progerin did not disrupt their colocalization (Figure S2B). As described above, progerin began to diffuse into the cytoplasm during prophase, whereas lamin A and lamin C remained at the lamina (Figure 1 and Figure S2). In HGPS cells, progerin was recruited to the chromosome mass and daughter nuclei much later than lamin A and lamin C in HGPS cells (Figure 1 and Figure S2C). In HGPS cells, the sequential distribution patterns of lamin A and lamin C were similar to those observed in normal cells. However, the kinetics of events were delayed in HGPS cells, from metaphase until cytokinesis, as indicated by the accumulation of lamin A and lamin C in the cytoplasm during cytokinesis (Figure 1 and Figure S2).

To further investigate the mitotic impact of progerin, we compared the distributions of progerin and lamin B1 (Figure 1C, 1D). In control cells, the lamin B1 rim staining became discontinuous at prophase during NE breakdown (Figure 1C). In metaphase, lamin B1 appeared diffused and granular throughout the cytoplasm (Figure 1C). In late anaphase, lamin B1 started to localize to the pole regions of the chromosomes [24]. In telophase, lamin B1 reformed a rim, and by cytokinesis, it was entirely engaged at the nuclear periphery, as previously reported [24] (Figure 1C).

During interphase, HGPS cells exhibiting low levels of progerin, lamin B1 was localized to the NE and nuclear blebs (Figure 1D). Similar to previous reports [25], when progerin accumulated in HGPS nuclei, lamin B1 levels were decreased, as indicated by the weak lamin B1 staining in the large, dysmorphic interphase nucleus (Figure 1D, arrowhead). During prophase, the lamin B1 rim became discontinuous, and large progerin aggregates were visible in the cytoplasm by prometaphase. From metaphase until early anaphase, both lamin B1 and progerin appeared granular and diffused throughout the cytoplasm in HGPS cells, and were only partially colocalized (Figure 1D). In late anaphase, small discrete structures of lamin B1 appeared around the chromosome regions, whereas progerin aggregates remained largely diffused in the cytoplasm (Figure 1D). During telophase, lamin B1 began to reform a rim that remained discontinuous until cytokinesis, and large amounts of lamin B1 aggregates persisted in the cytoplasm of HGPS daughter cells (Figure 1D). Collectively, these data indicate that in normal cells, lamin B1 is recruited to the NE and exhibits typical rim-like staining during cytokinesis. However, in HGPS cells, lamin B1 exhibited a delay in its recruitment that resulted in the accumulation of lamin B1 aggregates in the cytoplasm and its partial co-distribution with progerin at the end of mitosis in 18% of the cells with weak progerin signal and 98% of the cells with strong progerin signal (Figure S1).

### Dynamics of progerin and the inner NE proteins emerin and SUN1 in mitotic HGPS cells

**Figure 2:**
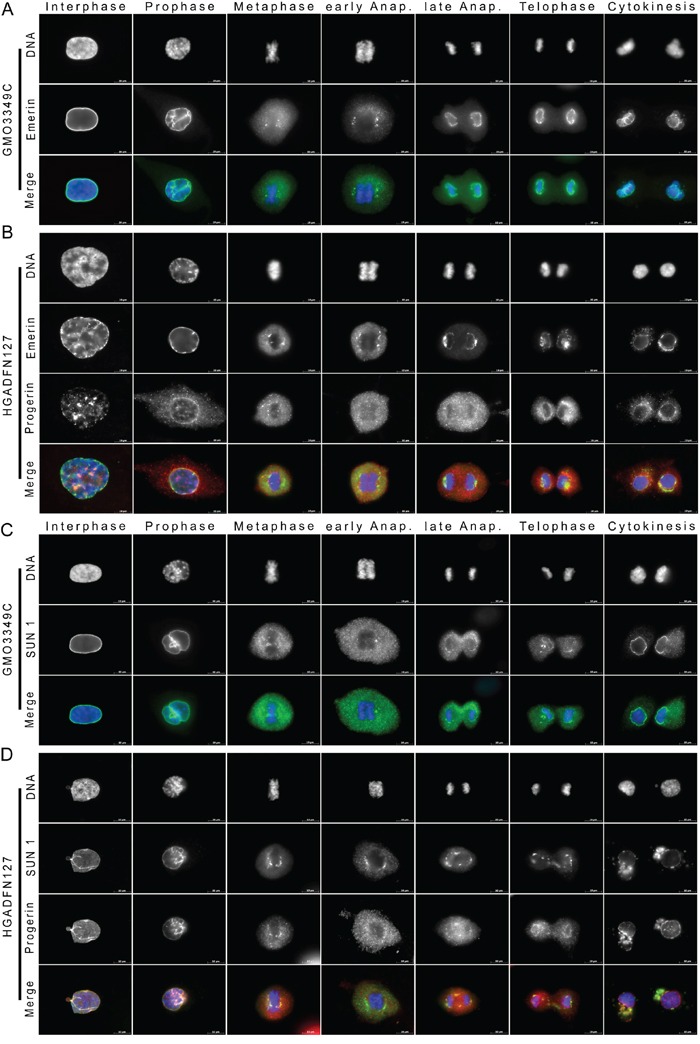
Emerin and SUN1 distribution are altered in mitotic HGPS fibroblasts **A.** Immunocytochemistry of control (GMO3349C) and **B.** HGPS (HGADFN127) using antibodies directed against emerin (green) and progerin (red) are shown as single images (n=19). Chromatin was stained with DAPI (blue). The triple merge signals are shown. Emerin, an INM protein, showed a rim-like staining during interphase that partially overlapped with the progerin signal during interphase. Typical emerin-rim staining was detected during telophase in control cells, whereas in HGPS cells, emerin-rim was faint and emerin aggregates colocalized with progerin in the cytoplasm. **C.** Immunocytochemistry using antibodies directed against SUN1 (green) and progerin (red) was performed on control (GMO3349C) and **D.** HGPS fibroblasts (HGADFN127), (n=25). SUN1 an INM protein was colocalized with progerin during interphase at the NE, at the core-region of metaphase chromosomes and during the subsequent mitotic stages. Large cytoplasmic SUN1- and progerin-positive aggregates were present during cytokinesis in HGPS cells. The triple merged signals are indicated. Scale bar, 10 μm.

Because the lamina components showed altered and delayed recruitment to the segregating chromosomes and daughter nuclei in HGPS cells, we next investigated the impact of progerin on NE dynamics and evaluated the repartitioning of emerin, an integral INM protein [26]. In normal cells, emerin showed a typical nuclear rim staining during interphase (Figure 2A). During prophase, the rim became discontinuous, and emerin became granular and diffused in the cytoplasm in late prophase. During metaphase, emerin remained partly granular in the cytoplasm, but clear emerin deposits were observed at the core region of metaphasic chromosomes until early anaphase (Figure 2A). These emerin membranous structures elongated to the periphery of the chromosomes until late anaphase, and by telophase, emerin had reformed a typical nuclear rim distribution in the daughter nuclei, indicating that the NE had reformed (Figure 2A). In normal cells, emerin was entirely recruited to NE during cytokinesis (Figure 2A).

During interphase, emerin staining showed some thickening at the NE periphery of HGPS nuclei in bright progerin-positive cells (Figure 2B). Emerin staining was discontinuous at sites where progerin was present in large aggregates (Figure 2B). Overall, the emerin and progerin distribution patterns were only partially overlapping during interphase and mitosis (Figure 2B). During prophase, progerin was visible in the cytoplasm, but most of the emerin remained at the periphery of condensing chromosomes. During metaphase, elongated emerin membrane structures appeared in the vicinity of the metaphasic chromosomes, whereas most of the progerin aggregates remained diffused in the cytoplasm (Figure 2B). From early anaphase until late anaphase, emerin elongated around the chromosomes and surrounded the daughter nuclei only during cytokinesis (Figure 2B), indicating that the recruitment of emerin to the NE was delayed in HGPS cells (Figure 2B). Hence, a large amount of progerin remained in the cytoplasm during cytokinesis, where it partly colocalized with emerin cytoplasmic aggregates. This result suggested that a fraction of cytoplasmic progerin was trapped by membrane-bearing emerin in the cytoplasm at the end of mitosis (Figure 2B). Emerin was mislocalized in approximately 25% of the mitotic cells with weak progerin signal and 97% of the cells with strong progerin signal (Figure S1).

Emerin is an interacting partner of lamin A/C [27]. In accordance with previous studies, in normal cells, a clear temporal correlation was observed between emerin and lamin A association with the reforming NE in telophase (Figure S3A) [28]. In HGPS cells, emerin and lamin A also co-repartitioned at discrete structures from metaphase until late anaphase, and their recruitment to the chromosome mass was delayed until cytokinesis (Figure S3B). Hence, the cytoplasmic aggregates of lamin A and emerin were superimposable during cytokinesis (Figure S3B). These results indicate that during late cytokinesis, cytoplasmic membranes bearing emerin remained associated with lamin A/C and progerin in HGPS cells.

To further explore NE dynamics during mitosis in HGPS cells, we evaluated the mitotic distribution of another lamin A/C-interacting partner, SUN1, a member of the linker of nucleoskeleton and cytoskeleton (LINC) complex [29].

In normal cells, SUN1 was localized to the NE in a typical rim-like staining (Figure 2C). During metaphase, SUN1 became dispersed in the cytoplasm and formed distinct structures in the vicinity of the metaphase chromosomes (Figure 2C). In early anaphase, SUN1 deposits appeared at the core region of the segregating chromatids, but was excluded from the chromosome mass (Figure 2C). In late anaphase, SUN1-positive membrane structures began to elongate around the chromosomes, and a rim-like staining pattern was observed by telophase (Figure 2C). During cytokinesis, most SUN1 was recruited to the nuclear periphery (Figure 2C).

In HGPS cells, the SUN1 signal was highly heterogeneous and reminiscent of the progerin staining (Figure 2D). Bright SUN1-positive nuclei corresponded to the most dysmorphic HGPS nuclei, which also harbored a strong progerin-positive signal. Dim SUN1-positive nuclei exhibited a weak to barely detectable progerin signal (Figure 2D). The non-homogeneous SUN1 staining we observed in HGPS cultures is similar to the results observed in previous studies [21, 30]. During metaphase, SUN1 accumulated at the core region of metaphase chromosomes and overlapped with progerin deposits (Figure 2D). It then began to elongate along the chromosome mass (Figure 2D). In late anaphase, in contrast to normal cells, SUN1 was excluded from the spindle midzone and extended into thick membrane structures above the spindle area, where it colocalized with progerin (Figure 2D). During telophase, SUN1 membrane patches were recruited with progerin to the segregated chromosomes (Figure 2D). During cytokinesis, in contrast to normal cells, large amounts of SUN1 remained in the cytoplasm, and only a thin SUN1 rim staining was detected in HGPS daughter cells (Figure 2D). Collectively, these data indicate that the spatiotemporal distribution of SUN1 during mitosis was dramatically altered when progerin was presence. SUN1 recruitment to the nuclear periphery was delayed by anaphase, and SUN1 largely co-distributed with progerin, indicating that progerin remained associated with SUN1 membrane structures in HGPS mitotic cells. SUN1 was mislocalized in an average of 27% of the mitotic cells with a weak progerin signal and an average of 98% of the cells with strong progerin signal (Figure S1).

### Dynamics of progerin and the ER protein calnexin in mitotic HGPS cells

The NE is a specialized subdomain of the ER that surrounds the nucleus as a double membrane [31]. Because the INM proteins emerin and SUN1 exhibit delayed recruitment to the chromatin periphery during telophase, we investigated the dynamic properties of the ER during mitosis by analyzing the repartition of calnexin, a calcium-binding protein that resides in the ER.

**Figure 3:**
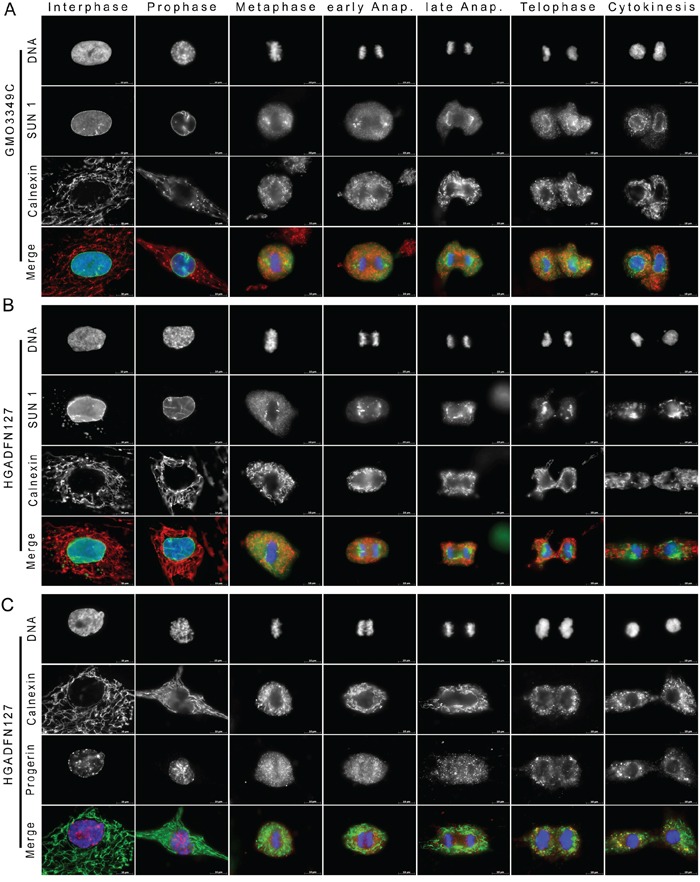
Large amount of SUN1 remains trapped in the ER at the end of mitosis in HGPS fibroblasts **A.** Immunocytochemistry of control (GMO3349C) fibroblasts was performed with anti-SUN1 (green) and anti-calnexin (an ER marker) (red) antibodies. Chromatin was stained with DAPI (blue). The triple merged signals are indicated. Scale bar, 10 μm, (n=8). In control cells, after NE breakdown during prophase SUN1 was colocalized with calnexin at the ER until being recruited to the nuclear periphery during telophase. **B.** HGPS (HGADFN127) fibroblasts were labeled with anti-SUN1 (green) and anti-calnexin (red) antibodies (n=8). In HGPS cells, in contrast to control cells, SUN1 cytoplasmic aggregates remained trapped in the ER during cytokinesis. **C.** HGPS HGADFN127 fibroblasts were labeled with anti-progerin (red) and anti-calnexin (green) antibodies (n=14). The progerin signal partially overlapped with the calnexin signal during the different stages of mitosis. Progerin cytoplasmic aggregates co-distributed with calnexin at the ER during cytokinesis.

In interphase control cells, calnexin was localized to the fibrous ER network that extended from the nuclear periphery toward the plasma membrane (Figure 3A). As the NE membrane retreated, SUN1 dispersed into the ER, and calnexin staining revealed a collapsed network that kept away from the condensing chromosomes (Figure 3A). During metaphase, the calnexin network formed a cage-like structure that partially colocalized with SUN1 around the metaphasic chromosomes, indicating that the NE was completely reabsorbed into the ER compartment. A few SUN1 membrane structures were located at the spindle pole sides of the metaphase chromosomes, but these did not include calnexin (Figure 3A). In control cells, the calnexin membrane network remained excluded from the mitotic spindle midzone until late anaphase (Figure 3A). Once the NE was reformed, as indicated by the SUN1 rim-like staining during telophase, the ER re-extended from the NE periphery throughout the cytoplasm (Figure 3A).

In HGPS cells, the ER-calnexin network staining was similar to control cells during the breakdown of the NE, and it remained excluded from the spindle area until late anaphase (Figure 3B). However, because SUN1 recruitment to the reforming NE was delayed, the ER-calnexin network remained disorganized and collapsed in the cytoplasm of daughter cells (Figure 3B). These data indicated that in HGPS cells, a large amount of SUN1 remained trapped within the ER during late cytokinesis.

To further validate the observation that progerin co-distributes with SUN1 in the ER during mitosis, we analyzed the distribution pattern of progerin in comparison to calnexin distribution (Figure 3C). Similar to the SUN1 distribution, progerin staining overlapped with calnexin staining in some areas of the ER from metaphase until anaphase (Figure 3C). Progerin aggregates were in close vicinity to calnexin staining during cytokinesis (Figure 3C).

Collectively, our observations indicate that components of the lamina (lamin A/C and lamin B1) and the INM (emerin and SUN1) were abnormally retained in the ER with progerin at the end of mitosis in HGPS cells. These findings also indicated that abnormal protein-membrane interactions occurred during metaphase and anaphase, and that these interactions interfered with the normal recruitment of these proteins to the reforming nuclear lamina and NE during late anaphase and telophase. Consequently, these nuclear proteins were found to be retained in the ER compartment after mitosis was completed. Notably, SUN1 distribution was the most highly disrupted in mitotic HGPS cells. The frequencies of the mitotic defects described in the mitotic profiling of the nuclear lamina and NE proteins were directly correlated with the levels of progerin that were present in HGPS cells (Figure S1). In all, 96% to 98% of the mitotic cells that exhibited a strong progerin signal displayed mislocalized nuclear proteins (Figure S1).

### Progerin and nuclear pore dynamics in mitotic HGPS cells

**Figure 4:**
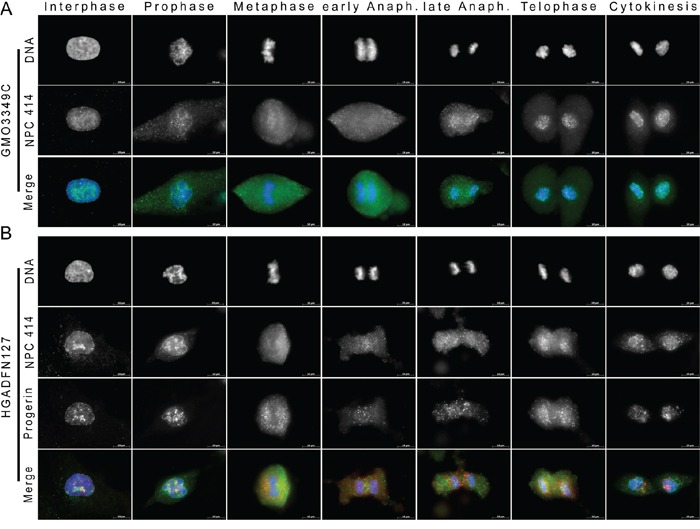
Recruitment of nuclear pores to the reforming NE is delayed in HGPS fibroblasts **A.** Immunocytochemistry using antibodies directed against NPC 414 (green) and progerin (red) was performed on control (GMO3349C) and **B.** HGPS (HGADFN127) fibroblasts. Chromatin was stained with DAPI (blue). The triple merged signals are indicated. Scale bar, 10 μm, (n=27). Whereas in control cells, NPCs were recruited to the NE during telophase, in HGPS cells, NPCs were only detected during late telophase to cytokinesis. NPCs appeared clustered at the NE during cytokinesis in HGPS daughter nuclei.

We investigated the progerin distribution relative to nuclear pore complexes (NPCs), which require NE at the chromosome periphery for their reinsertion during mitosis (Figure 4). We stained NPCs using the monoclonal antibody 414, which is directed against nucleoporin. During interphase, NPCs signal appeared as punctate dots distributed throughout the nucleus in both the control and the HGPS cells (Figure 4). During metaphase, both the NPCs and progerin showed a diffused and granular distribution in the cytoplasm, and they did not colocalize (Figure 4). In both cell types, NPCs remained throughout the cytoplasm until anaphase. During late anaphase, in normal cells, NPCs began to be recruited to the newly reforming NE, and a large number of NPCs remained visible in the cytoplasm (Figure 4A). During telophase, most of the NPCs surrounded the chromosome periphery and formed a thin, dotted, rim-like structure (Figure 4A). A typical NPC rim was observed by cytokinesis in normal cells (Figure 4A). In contrast, in HGPS cells, numerous NPCs remained in the cytoplasm during telophase, and the NPCs at the NE showed a discontinuous and uneven distribution pattern (Figure 4B). Large NPC aggregates or clusters remained in some areas of the NE during telophase and cytokinesis (Figure 4B). These NPC clusters were also observed in the vicinity of the progerin signal (Figure 4B). These results indicated NPCs recruitment was delayed during telophase in HGPS cells, and this finding is consistent with the delay in recruitment that was observed for the INM proteins emerin and SUN1. This observation suggested that the alterations in NE reformation during telophase that were observed in HGPS cells might be responsible for the delayed reinsertion of NPCs. The uneven insertions of NPCs in the NE might be responsible for the formation of NPC clusters, which are one of the hallmarks of interphase HGPS cells [7, 8]. We determined that an average of 9% of the mitotic cells with a weak progerin signal and 97% of the cells with a strong progerin signal exhibited mislocalized NPCs (Figure S1).

### Scoring of chromatin defects during the progression of mitosis in HGPS cells

**Figure 5:**
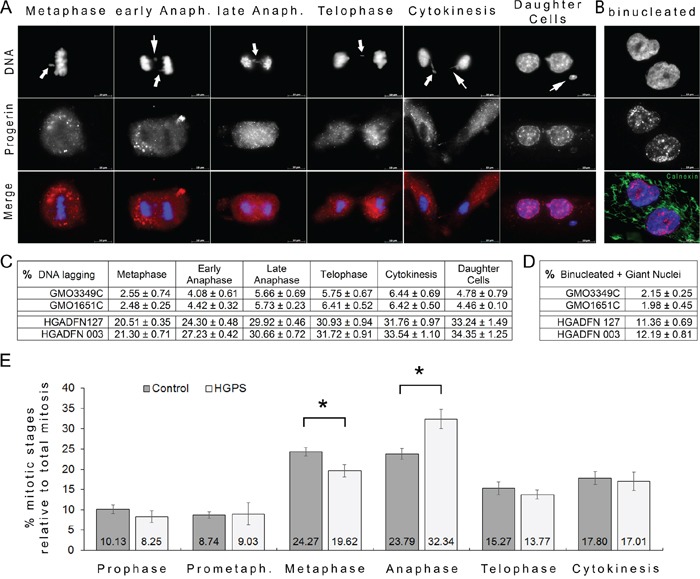
Frequent chromatin lagging occurred in mitotic HGPS cells **A.** HGPS (HGADFN127) fibroblasts were labeled with DAPI (DNA, blue), and anti-progerin antibody (red). Representative images exhibiting chromatin lagging during the different stages of mitosis are shown. Arrowheads indicate the position of chromatin lagging. **B.** Binucleated cells were labeled with DAPI (blue) and calnexin (green); a representative image is shown. Double merged signals for progerin with DNA or calnexin with DNA are shown. Scale bar, 10 μm. **C.** The numbers of cells exhibiting lagging chromatin at different stages of mitosis were scored as described in the Materials and Methods in control (GMO3349C, GMO1651C) and HGPS cells (HGADFN127, HGADFN003). The average mitotic index was on average 1.40% in control cultures and 0.94% in HGPS cultures. Quantification of mitotic cells exhibiting lagging chromatin was scored for each stage of mitosis and the percentage relative to the total number of mitotic cells present in control or HGPS cultures was determined as indicated. A total number of 5217 cells were screened in control and 4976 in HGPS cultures out of 6 independent cultures for each control and HGPS cell lines. The values are reported as means ± SD and evaluated using Student's t-test. **D.** The percentage of binucleated cells and cells with giant nuclei were scored by direct count of a total of 1559 control (GMO3349C, GMO1651C) cells and 2130 HGPS (HGADFN127, HGADFN003) cells derived from 3 independent cultures. Data are presented as means ± SD and evaluated using Student's t-test. **E.** In parallel to the evaluation described in (C), the numbers of cells found at a specific mitotic stage were scored and determined as described in (C). Mitotic HGPS cells were found to be increased during anaphase.

The delayed recruitment of nuclear components to the chromosome mass during mitosis and the observation of binucleated HGPS cells suggested that distinct dysfunctions occur during mitotic exit, including chromosome segregation errors or cytokinesis failures, in these cells [19]. To understand this process better, we determined the binucleated cell index scores, which are indicative of cytokinesis defects. These scores were markedly increased in HGPS cells (Figure 5), and as previously reported [19]. We next scored the occurrence of lagging chromatin during the different stages of mitosis to track potential early mitotic defects in chromosome segregation (Figure 5A, 5B, 5C). Aberrant chromatin positioning was readily detectable during metaphase and during the subsequent mitotic stages until cytokinesis, when chromatin deposits were frequently observed at the cleavage furrow (Figure 5A). This chromatin profiling provided us with an overview of the global mitotic patterns and temporal perturbations that occur in HGPS cells as a result of the presence of progerin, because normal cells rarely exhibited these chromatin alterations during mitosis (Figure 5B, 5C). The difference in the mitotic indexes of control (average: 1.41%) and HGPS (average: 0.95%) cells taken from asynchronous cultures from passages 10 to 16 indicated that the control cells exhibit 1.5-fold more mitotic cells than the HGPS cultures. This observation indicates that HGPS cells grow more slowly than normal cells, as reported previously [7, 8].

To identify a potential point of delay in a particular stage in mitosis, we scored the percentages of cells that were in different mitotic stages based on DNA and α-tubulin labeling (Figure 5E). We found that an increased proportion of cells were in anaphase in the HGPS cultures. These results demonstrated that HGPS cells accumulated during this stage, which suggested a slower exit or transition to telophase (Figure 5E) than occurs in normal cells. Collectively, these data indicate that progerin induces alterations in chromosome segregation as early as metaphase by delaying the exit from anaphase, which ultimately results in an increase in the proportion of binucleated HGPS cells.

**Figure 6:**
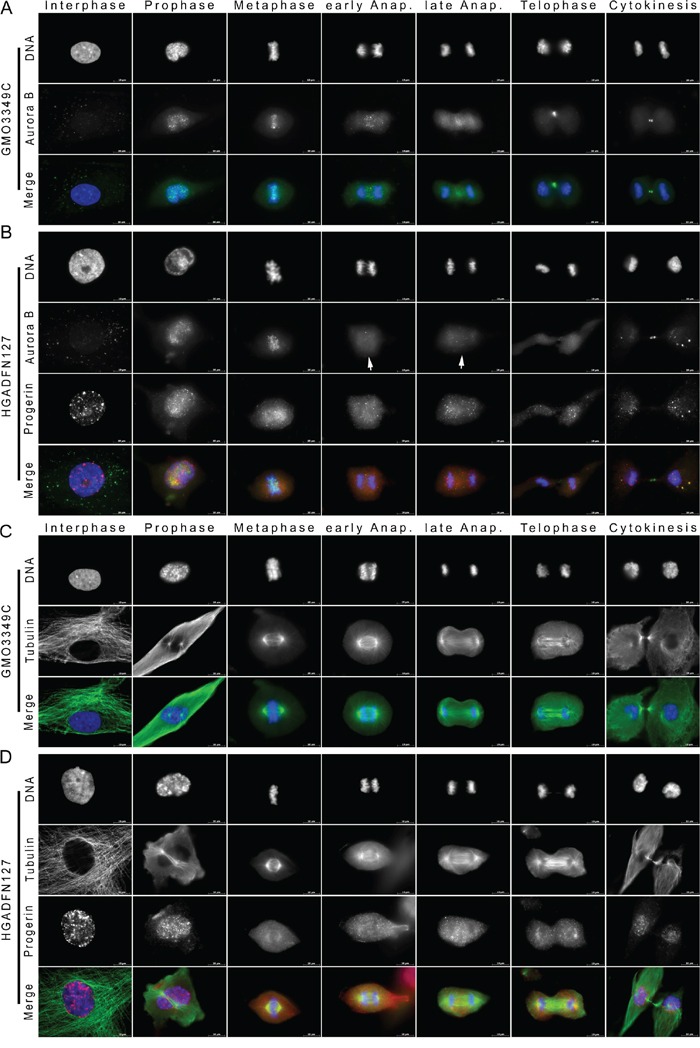
Aurora kinase B distribution is altered in mitotic HGPS cells **A.** Immunocytochemistry was performed on control (GMO3349C) and **B.** HGPS fibroblasts (HGADFN127) using antibodies directed against progerin (red) and Aurora B (green). Representative images from interphase and different mitotic stages are shown (n=17). Merged images correspond to the triple signals including DAPI (blue). Arrowheads indicate loss of Aurora B signal from the spindle midzone during anaphase in HGPS cells. **C, D.** Immunofluorescence labeled control (GMO3349C) and HGPS (HGADFN127) cells for the detection of progerin (red) and α-tubulin (green) at different stages of mitosis are shown (n=7). No significant changes in the distribution of microtubules were observed in mitotic HGPS cells. Scale bar, 10 μm.

To further investigate the possibility of an anaphase delay in HGPS cells, we monitored the distribution of Aurora B kinase (Figure 6A, 6B), an essential chromosome separation checkpoint that regulates the anaphase-telophase transition by forming a midzone gradient in anaphase cells [32]. In normal cells, Aurora B was recruited to the chromosome mass during metaphase (Figure 6A). As shown previously [33], Aurora B relocated to the spindle midzone during anaphase and telophase and to the cleavage furrow during cytokinesis (Figure 6A). In brightly progerin-positive HGPS cells, Aurora B localized with the metaphase chromosomes, but it became diffused during anaphase without showing a detectable accumulation in the midzone or at the cleavage furrow during cytokinesis (Figure 6B). Defects in Aurora B repartition and gradient formation during anaphase were observed in 12% of the cells that exhibited weak progerin signal and 95% of the cells that exhibited strong progerin signal (Figure S1). These data indicated that progerin induced a delay in cell cycle progression during the anaphase-telophase transition.

To determine the molecular mechanisms that underlie the defects in chromosome segregation and the increased rate of aneuploidy in HGPS cells, we compared progerin and microtubule distribution patterns during mitosis (Figure 6C, 6D). Whereas progerin localized at discrete sites close to the spindle pole side of the metaphase chromosome plate, no obvious abnormalities were observed in spindle formation or elongation during metaphase and anaphase (Figure 6C, 6D).

### Dynamics of progerin and the centromeric proteins CENP-F and CENP-E in mitotic HGPS cells

**Figure 7:**
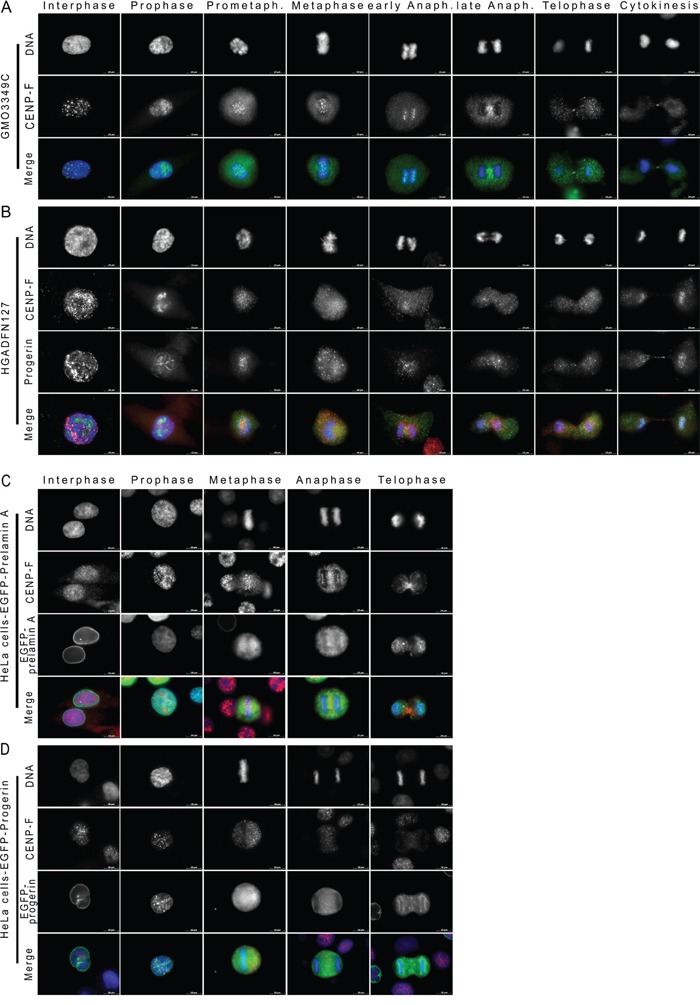
CENP-F is delocalized from kinetochores during metaphase in HGPS cells Immunocytochemistry was performed on **A.** control (GMO3349C) and **B.** HGPS fibroblasts (HGADFN127) using antibodies directed against CENP-F (green) and progerin (red). Representative images of mitotic stages are shown (n=18). Arrowhead indicates loss of CENP-F at metaphase kinetochores in HGPS cells. Merged images correspond to the triple signals including DNA (blue). **C.** Images of transfected HeLa cells with cDNA constructs encoding GFP-prelamin A (green) or **D.** GFP-progerin (green) labeled with anti-CENP-F (red) and DNA (blue) are shown. Arrowhead indicates loss of CENP-F at metaphase kinetochores in HeLa transfected with GFP-progerin construct. Scale bar, 10 μm (n=8).

Accurate chromosome segregation during mitosis is governed by kinetochores and spindle microtubules [34]. Therefore, we investigated the distribution patterns of two centromeric proteins, CENP-F and CENP-E, which are expressed during mitosis and are transiently localized to kinetochores during mitosis [35]. In normal cells, during interphase, CENP-F formed part of the nuclear matrix, and its signal was increased in cells that were in G2 phase (Figure 7A). Upon entry into prometaphase, a fraction of the CENP-F was released into the cytosol, and a sub-fraction was detected at the kinetochores until early anaphase (Figure 7A), as was previously reported [36]. During late anaphase, CENP-F was released from the kinetochores and it re-localized to the spindle midzone (Figure 7A). During telophase, CENP-F became diffused in the cytoplasm, and its signal had decreased by the end of mitosis (Figure 7A).

In HGPS cells, during interphase and G2, CENP-F appeared as small aggregates throughout the nucleus and at the NE (Figure 7B). Although CENP-F was in the vicinity of progerin aggregates, these proteins did not colocalize (Figure 7B). In HGPS cells with a strong progerin signal, CENP-F was reduced at the kinetochores during prophase, and in contrast to normal cells, CENP-F was no longer recruited to the kinetochores during metaphase and early anaphase in 97% of the HGPS cells (Figure 7B and Figure S1). CENP-F was not clearly detectable at the spindle midzone during anaphase, and it remained diffused throughout the cytosol (Figure 7B). In mitotic HGPS cells that exhibited barely detectable levels of progerin, the distribution of CENP-F was similar to that observed in normal cells (Figure S1). Thus, depending on the level of progerin in HGPS cells, CENP-F loses its ability to be recruited to the kinetochores during metaphase, and it therefore remains dispersed in subsequent mitotic stages (Figure 7B).

To further verify that progerin elicits the displacement of CENP-F from metaphase kinetochores, we transfected HeLa cells with either GFP-prelamin A or GFP-progerin constructs (Figure 7C, 7D). While no significant changes were observed in HeLa cells that were transfected with GFP-tagged prelamin A (Figure 7C), CENP-F became delocalized during metaphase and anaphase in HeLa cells that expressed GFP-tagged progerin (Figure 7D). Collectively, these findings suggest that progerin competes with CENP-F and thereby prevents CENP-F from binding to the kinetochore-microtubule interface during metaphase and anaphase.

Next, we examined another kinetochore protein, CENP-E, in mitotic HGPS cells (Figure S4). CENP-E showed a similar pattern of localization in mitotic control and HGPS cells (Figure S4). However, the CENP-E signal was rapidly reduced during anaphase in HGPS cells (Figure S4). Hence, the CENP-E signal at the spindle midzone in late anaphase was reduced, and its concentration was decreased at the cleavage furrow during cytokinesis. These data indicated that CENP-E levels decreased more rapidly after the onset of anaphase in HGPS cells than in normal cells (Figure S4).

**Figure 8:**
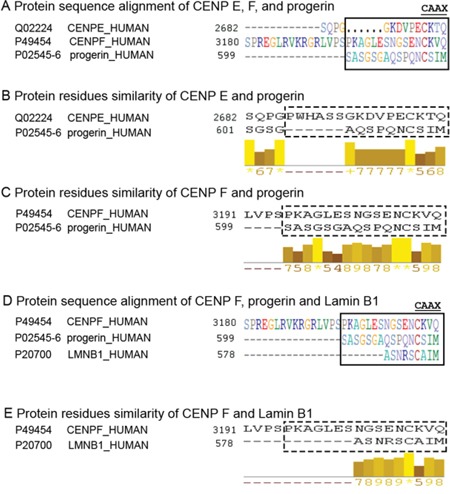
Progerin C-terminal sequence is similar to CENP-F terminus The PSI-BLAST [56] alignment tool (accessed through PredictProtein (www.predictprotein.org) [57] was used to align a family of related sequences, and the results revealed that progerin has a sequence similar in its C-terminus to CENP-F and CENP-E. All sequences were taken from UniProt [58]. **A.** The CAAX farnesylation motif implies the observation of the following four consecutive residues at the C-terminus: cysteine-aliphatic-aliphatic-any. Aliphatic amino acids are G, A, V, L, I, P (partial). The alignment of the three C-termini shows that the CAAX is not strictly contained in CENP-E and CENP-F. However, both contain CXAX and both are known to be farnesylated suggesting that CXAX may also indicate farnesylation [47]. **B.** The optimal alignment of the 16 final residues between CENP-E and progerin show a gap of six amino acids in CENP-E. The standard interpretation is that progerin might have a longer loop in this region. **C.** The optimal alignment of the 16 final residues between CENP-F and progerin do not show any gap at the end. The bars with values ranging from 0 to 10 indicate sequence similarity: the greater the value the greater the similarity according to the biophysical features of amino acids. The top value 10 (indicated by a star in the online abbreviation) marks identical residues. Progerin's 12 C-terminal residues have significant sequence similarity to the C-terminus of CENP-F; this similarity extends beyond the CAAX motif. **D.** The alignment of the C-termini of CENP-F, progerin and lamin B1 (LMNB1). **E.** The optimal alignment of the 16 final residues between CENP-F and Lamin B1 shows 9 amino acids in lamin B1 with some degree of similarity.

Both the CENP-E and the CENP-F protein harbor a CAAX motif at their carboxyl-terminal end, and similarly to progerin, they are also farnesylated [37]. Using Predict Protein software (https://www.predictprotein.org) [38], the protein sequences of CENP-E, CENP-F and progerin were compared. They showed some sequence similarity at their carboxyl-terminal end. A total of 16 amino acids (aa) in CENP-F aligned without gaps with progerin, but only 10 aa of CENP-E aligned with progerin (Figure 8A). Similarity scores for pairwise residue comparisons are provided and these were based on the physicochemical properties of each amino acid [38]. The longer the stretch of residues with a score above 5, the higher is the likelihood that the compared peptides share sequence similarities. Using this approach, the progerin terminal sequence showed a stronger resemblance to the CENP-F than to the CENP-E terminal sequence (Figure 8B, 8C). Analysis of the lamin B 1 sequence showed that only 9 residues aligned with CENP-F terminal sequence (Figure 8D, 8E). These observations suggest that the terminal end of progerin might more efficiently compete and/or prevent CENP-F protein-protein interactions at the kinetochore-microtubule interface. Collectively, these data suggest a potential link between progerin and genomic instability, which is one of the hallmarks of HGPS cellular phenotypes.

Next, to determine whether progerin-induced defects in mitosis might cause HGPS cells to enter premature senescence, we performed a senescence detection assay using β-galactosidase staining [39]. The proportion of senescent cells and the number of binucleated cells that were positive for senescence associated β-galactosidase staining were higher in the HGPS cultures than in normal fibroblast cultures (Figure S5). These findings indicate that binucleated HGPS cells have a tendency to enter senescence.

### Changes in nuclear protein status in HGPS cells

**Figure 9:**
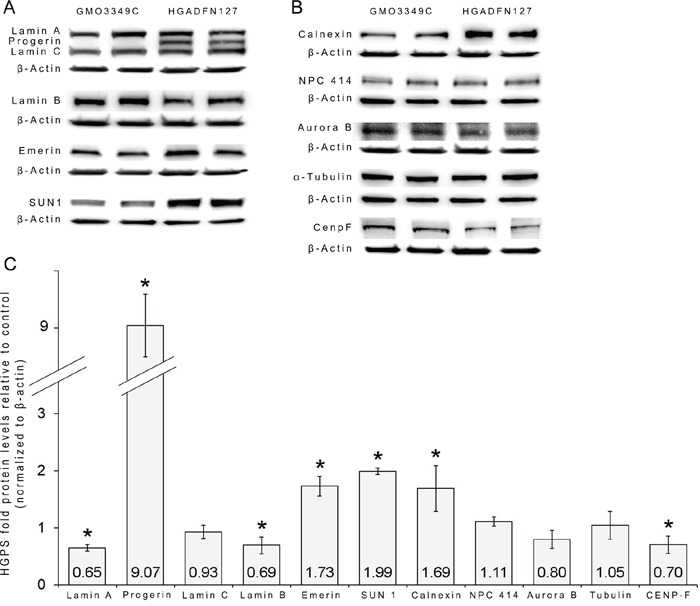
Status of the nuclear proteins in HGPS cells **A.** Representative Western blots are shown for lamin A/C, progerin, lamin B1, emerin, SUN1, and β-actin in total cell extracts that were isolated from control and HGPS cultures. **B.** Representative Western blots for calnexin, NPC 414, Aurora B, CENP-F, α-tubulin and β-actin are shown. **C.** Quantifications are shown for lamin A, progerin, lamin C, lamin B1, emerin, SUN1, calnexin, NPC 414, Aurora B, CENP-F and α-tubulin levels after normalization to β-actin as fold-changes relative to the levels of control cells (n=8; *P < 0.05, Student's t-test). Unsynchronized HGPS cells showed increased levels of progerin, SUN1, emerin and calnexin compared to control cells. Lamin B, Aurora B and CENP-F levels were reduced in unsynchronized HGPS cells than in control cells.

Western blot analyses of unsynchronized fibroblast cultures (Figure 9) showed that lamin A levels were lower in HGPS cells than in control cells. The major difference between the normal and HGPS cells was the presence of a high amount of progerin in the HGPS cells, which caused severe alterations in the statuses of A-type lamins (Figure 9A, 9C). The levels of the INM protein emerin and SUN1 were increased in HGPS cells, with the SUN1 level being approximately 2-fold higher in HGPS cells than in normal cells (Figure 9A, 9C). Remarkably, the level of calnexin, an ER marker, was also increased in HGPS cells (Figure 9B, 9C). Lamin B1, Aurora B and CENP-F were reduced in unsynchronized HGPS cells (Figure 9A, 9B, 9C). Collectively, these data indicate that the presence of progerin in HGPS nuclei elicits noticeable changes in the levels of INM proteins, with the most significant change occurring in the level of SUN1 (Figure 9C).

## DISCUSSION

To date, studies of HGPS have focused primarily on nuclear abnormalities during interphase, and only a few attempts to localize progerin during mitosis have been performed using HeLa cells transfected with plasmids encoding GFP-progerin [19-21]. We investigated the effects of endogenous progerin on the dynamics of mitosis in unsynchronized HGPS fibroblast cultures.

### Progerin-dependent mitotic defects in HGPS cells

The results of this study show that progerin induces defects in the reformation of the NE, which causes components of the lamina and the inner NE to be retained in the ER compartment during cytokinesis. In HGPS cells, progerin formed aggregates in the cytoplasm shortly after the breakdown of the NE during prophase. A few progerin deposits were located close to metaphase chromosomes at the side of the spindle poles. The majority of progerin aggregates were colocalized with SUN1 membrane structures, as was previously reported [21]. Hence, in HGPS cells, SUN1 recruitment to the nuclear periphery during telophase was markedly delayed, and a large amount of SUN1 that was associated with progerin remained in the ER compartment during late cytokinesis. In contrast to earlier observations using HeLa cells that were transfected with GFP-progerin plasmids, we found that lamin A/C, lamin B1 and emerin were only partially co-distributed with progerin from metaphase until telophase in HGPS cells [19, 20]. In agreement with these studies, we found that progerin cytoplasmic aggregates colocalized with the lamin A/C and emerin that remained trapped in the ER during late cytokinesis [19, 20]. Collectively, these findings suggest that progerin co-localization with SUN1 membrane structures during metaphase and lasts until telophase and may prevent the proper sorting of the NE from the ER during NE reformation. This may consequently cause a change in the temporal order of the recruitment of nuclear lamina components up to the end of mitosis in HGPS cells. A similar delay in the recruitment of the nuclear pores to the NE during reformation was observed in HGPS cells. Nuclear pores at the periphery of the newly formed nuclei appeared as patches or clusters, which were reminiscent of the clustering of the nuclear pores observed in interphase HGPS nuclei [7, 8]. Consistent with this observation, SUN1 depletion or overexpression was previously shown to induce nuclear pore clustering [40].

We also found that SUN1 levels were not homogeneous in all cells [21, 30]. The most dysmorphic HGPS nuclei were brightly labeled by anti-SUN1 and anti-progerin antibodies, indicating that SUN1 accumulated concomitantly with the build-up of progerin in HGPS cells. These bright progerin- and SUN1-positive HGPS nuclei corresponded to nuclei that showed reduced lamin B1 staining [25]. This novel nuclear staining signature designates a subpopulation of HGPS cells that are quiescent and/or senescent [23, 41]. The accumulation of progerin was also correlated with an increase in the frequency of binucleated HGPS cells that were positive for senescence β-galactosidase staining, which is a late marker of senescence [39]. In contrast, proliferative HGPS cells exhibited low to barely detectable levels of progerin and had similar levels of SUN1 as observed in normal cells. These results indicate that high levels of progerin might predispose cells to undergo premature cellular senescence.

### Progerin depletes CENP-F from metaphase kinetochores

In the present study, we showed that a high frequency of lagging chromatin was observed throughout metaphase until cytokinesis in HGPS cells, in accordance with previous studies [19]. The presence of lagging chromatin was always correlated with the presence of a detectable amount of progerin, indicating that the lagging chromatin frequency increased proportionally with the level of progerin in HGPS cells. Hence, this defect ultimately caused a marked increase in the numbers of binucleated cells and giant nuclei in HGPS cultures, whereas such errors rarely occurred in normal mitotic cells [19]. Because mitotic HGPS cells displayed no detectable changes in mitotic spindle formation, we hypothesized that lagging chromatin might originate from a weaker attachment of kinetochores to the spindle microtubules.

Kinetochores are assembled at the periphery of centromeric chromatin and are required to segregate chromosomes [42]. Some kinetochore proteins are constitutively localized to the chromosome centromere, whereas others assemble at kinetochores beginning in prophase and then leave the kinetochores at the end of mitosis [34]. Two of these proteins, the centromere proteins CENP-E and CENP-F, were of interest because of several of their properties. CENP-E is a motor protein that contains a long coiled-coil domain and a globular tail domain [43]. CENP-F is a large protein that contains a central coiled-coil domain and two microtubule-binding domains, one at each terminus [44, 45]. Both proteins are farnesylated and cell cycle-regulated [37, 46, 47].

These properties prompted us to evaluate the effects of progerin on CENP-E and CENP-F localization during mitosis. In normal cells during prometaphase and metaphase, CENP-E and CENP-F are localized on kinetochores [47, 48]. In HGPS cells, the distribution of CENP-E was similar to that observed in normal cells, but it exhibited a delay in relocation to the spindle midzone during anaphase. In contrast, CENP-F was present on kinetochores in prometaphase, but was absent from kinetochores in metaphase and remained diffused in the cytoplasm until the end of mitosis in HGPS cells.

To investigate why CENP-F was mislocalized in mitotic HGPS cells, we performed a protein sequence comparison using Predict Protein software [38]. The comparison of the carboxyl-terminal domains of CENP-E, CENP-F and progerin showed that they all end with a CAAX motif, which is a target for farnesylation [20, 37]. The last 16 amino acid residues of progerin showed a certain degree of sequence similarity with the CENP-F terminal end. We therefore, hypothesized that progerin's 16-residue sequence, which includes the farnesyl group, might compete with CENP-F for its proper location at the metaphase kinetochore-microtubule interface. Although further studies are needed to dissect the modalities of such a putative interaction, we propose that CENP-F could be a target of progerin during mitosis. Several lines of evidence support this hypothesis. (1) In this study, HeLa cells transfected with a GFP-progerin plasmid exhibited mislocalization of CENP-F during mitosis, as was observed in mitotic HGPS cells. (2) Previous studies have shown that silencing CENP-F in cells induced chromosome alignment defects [49, 50]. (3) Alterations in chromosome maintenance have been observed in cells treated with FTIs [37, 51, 52]. Intriguingly, these studies used FTIs and showed a similar pattern of delocalization from metaphase kinetochores for CENP-F [37, 51, 52], indicating that farnesylated CENP-F plays an important role in the maintenance of kinetochore-microtubule attachments during chromosome segregation.

In light of this observation, it is important to mention that HGPS pathology has been attributed in part to the farnesylation of progerin [4, 14, 18, 53] and that FTIs are currently being used in clinical trials for children with HGPS [1] (PRF: http://www.progeriaresearch.org). Whereas FTIs appear to ameliorate the disease condition *in vivo* [1], they induce a loss of bipolar spindle formation, donut-shaped nuclei and binucleated cells at the cellular level [19, 25, 54]. It remains to be investigated whether CENP-F is a common denominator between progerin and the FTI-induced defects in chromosome maintenance. However, we propose that the farnesylated carboxyl-terminus of progerin might dislodge CENP-F from its normal location at the metaphase kinetochore-microtubule interface, which weakens protein-protein interactions, and this may lead to defective chromosomal maintenance, increased lagging chromatin and aneuploidy in HGPS cells. This molecular mechanism could provide a link between progerin and genomic instability, and it opens a new area for future investigations and therapeutic strategies.

## MATERIALS AND METHODS

### Cell culture

Dermal fibroblasts from subjects with HGPS were obtained from the Progeria Research Foundation (www.progeriaresearch.org). The following fibroblasts were used: HGADFN003, HGADFN127, HGADFN155, and HGADFN188. Control dermal fibroblasts were obtained from the Coriell Institute for Medical Research (Camden, NJ). The following cell lines were used: GMO1651C, GMO3349C, GMO1852, and GMO3348E. All cell lines were used between passage 10 and 16 to screen mitotic cells. The Institutional Review Board of the Technische Universität München (TUM) approved the use of human cells in this study.

Cells were cultured in DMEM containing 15% fetal bovine serum, 1% glutamine and 1% penicillin/streptomycin. Cells were subcultured at 80% confluency to maintain the cultures in growth phase, as previously described [15].

### Western blot analysis

Cell pellets were extracted in Laemmli sample buffer (Bio-Rad), and Western blot analysis was performed as previously described [23]. The membranes were incubated with the following primary antibodies: anti-lamin A/C (mab636, MA3-1000, Thermo-Fisher), anti-progerin (rabbit monoclonal, 0.1 μg/mL) [22], anti-lamin B1 (M-20, sc-6217, Santa Cruz Biotechnology, 1/50), anti-emerin (4G5, NCL-Emerin, Leica Biosystems, 1/500), anti-SUN1 (Ab1, HPA008346, Sigma, 1/500), anti-calnexin (AF18, ab31290, Abcam, 1/500), anti-NPC (Nup414, ab50008, Abcam, 1/600), anti-NPC (Mab414, BioLegend MMS-120P, 1/2000), anti-Aurora B (ab3609, Abcam, 1/100), anti-α-tubulin (B-5-1-2, T5168, Sigma, 1/3000), anti-β-3-tubulin (TU-20, MA1-19187, Thermo Fisher, 1/2000), anti-CENP-F (14C10/1D8, ab90, Abcam, 1/300), anti-CENP-F (ab5, Abcam, 1/300) and anti-β-actin (Sigma-Aldrich, 1/5000). The membranes were then washed and incubated with the corresponding secondary antibodies coupled to horseradish peroxidase (Jackson ImmunoResearch Laboratories). Proteins were visualized using a chemiluminescence detection system (ECL substrate; Bio-Rad). Signals were analyzed using image lab software (Bio-Rad). Protein signals were quantified and normalized to β-actin.

### Immunocytochemistry

Control and HGPS fibroblasts were grown directly on coverslips. Cells were fixed 48 hours after splitting in 100% methanol at −20°C or in 3% paraformaldehyde (PFA) for 10 min at room temperature. Cells were then permeabilized in PBS containing 1% Triton X-100 for 10 min and further processed for immunohistochemistry, as previously described [23]. The following primary antibodies were used in this study: anti-progerin (rabbit monoclonal antibody, 1 μg/mL) [22], anti-lamin A (133A2, ab8980, Abcam, 1/500), anti-lamin A (L1293, Sigma, 1/700), anti-lamin C (ab125679, Abcam, 1/500), anti-lamin B1 (M-20, sc-6217, Santa Cruz Biotechnology, 1/50), anti-lamin B1 (8D1, sc-56144, Santa Cruz Biotechnology, 1/50), anti-emerin (4G5, NCL-Emerin, Leica Biosystems, 1/500), anti-SUN1 (Ab1, HPA008346, Sigma, 1/500), anti-calnexin (AF18, ab31290, Abcam, 1/500), anti-nuclear pore complexes (Nup414, ab50008, Abcam, 1/600), anti-nuclear pore complexes (Mab414, BioLegend MMS-120P, 1/2000), anti-Aurora B (ab3609, Abcam, 1/100), anti-α-tubulin (B-5-1-2, T5168, Sigma, 1/3000), anti-CENP-E (1H12, ab5093, Abcam, 1/500), anti-CENP-F (14C10/1D8, ab90, Abcam, 1/300), and anti-CENP-F (ab5, Abcam, 1/300), and anti-CENP-F (14C10/1D8, ab118857, Abcam, 1/300).

The secondary antibodies were affinity-purified Alexa Fluor 488 or 555 antibodies (A21202, A31570, A21206, A31572, A21206, A11055, and A21432 Life Technologies). All samples were also counterstained with DAPI in Vectashield mounting medium (Vector Inc., VEC-H-1200). Images were acquired using an Axio Imager D2 fluorescence microscope (Objective X63 oil, Carl Zeiss) and a Zeiss AxioCam MRm or by confocal microscopy on a Zeiss LSM 510 META confocal laser scanning system (Carl Zeiss).

All of the antibodies used in this study were tested using western blot analysis to verify their specificities. All antibodies were tested using single immunofluorescence staining in both control and HGPS cells. In addition, we determined the spatiotemporal distribution of single nuclear proteins to prevent any misinterpretation as a result of possible cross-reaction between the antibodies.

For statistic evaluations, cells from 3 normal and 3 HGPS cell lines in passages 10 to 16 were grown on coverslips. In an average of 3000 cells per coverslip, an average of 42 mitotic cells was observed in the control cultures, while an average of 27 mitotic cells was observed in the HGPS cultures. The mitotic index in the control cultures was 1.409% ± 0.021, while in the HGPS cultures the mitotic index was 0.949% ± 0.049.

To acquire good images and to perform statistical analyses of cells at different stages of mitosis, a minimum of seven independent double immunostaining experiments were required for each cell line that was analyzed (3 controls and 3 HGPS). The number of experimental repeats for each double immunodetection is indicated in legend caption.

### Mitotic index and mitotic chromosome lagging index

The mitotic index was determined by direct counting in control (GMO1651C and GMO3349C) and HGPS fibroblasts (HGADFN003 and HGADFN127) that were fluorescently labeled with anti-α-tubulin antibodies. DNA was labeled with DAPI. Cells were directly scored using immunofluorescence microscopy. Approximately 21,106 control cells were counted, and 19,277 HGPS cells were counted. These counts were derived from 3 different cultures of two different controls and two HGPS cell lines to determine the proportion of mitotic cells. Mitotic cells were scored as prophase, prometaphase, metaphase, anaphase or telophase based on DNA and α-tubulin labeling in both normal and HGPS cells. Similarly, we scored the incidence of lagging chromatin that was observed during the different stages of mitosis in the control and HGPS cells. For all of the experiments, the results are presented as the mean+/− SD. Comparisons were performed using Student's t-test.

### HeLa cell transfection

pEGFP-C1-progerin and pEGFP-C1-prelamin A constructs were kindly provided by Dr. Howard J. Worman (Columbia University of New York) and have been previously described [55]. HeLa cells were seeded on glass coverslips at an initial density of 1.5 × 10^5^ and incubated at 37°C for 24 hours. The cells were then transfected with 2 μg of the designated plasmids using FuGENE^®^HD Transfection Reagent (Promega) according to the manufacturer's instructions. After 24 or 48 hours of transfection, cells were fixed in 3% PFA at room temperature for 15 min followed by methanol fixation for 3 min at −20°C. Cells were blocked in 10% FBS in PBS for 30 min and subjected to immunofluorescence staining as described above.

### Senescence detection using β-galactosidase staining

HGPS cells grown on coverslips were washed once in PBS, fixed in 2% formaldehyde/0.2% glutaraldehyde for 5 minutes, and then stained overnight at 37°C with a solution containing 1 mg/mL 5-bromo-4-chloro-3-inolyl-β-galactosidase in dimethylformamide (20 mg/mL stock), 5 mM potassium ferricyanide, 150 mM NaCl, 40 mM citric acid/sodium phosphate, and 2 mM MgCl2, at pH 6.0 [39]. The cells were then washed twice with PBS and further processed for indirect immunostaining using anti-α-tubulin. DNA was stained with DAPI (Vector Inc., VEC-H-1200). Images were acquired using an Axioskop 2 mot plus fluorescence microscope (Objective X20 and X50, Carl Zeiss). ImageJ was used to merge single channel images. Blue β-galactosidase staining is presented as black signal in the bright-field image.

## SUPPLEMENTARY FIGURES


