# Supplementary material for: Progerin impairs chromosome maintenance by depleting CENP-F from metaphase kinetochores in Hutchinson-Gilford progeria fibroblasts
Source: Oncotarget. 2016 Mar 22;7(17):24700–18. doi: 10.18632/oncotarget.8267 (PMC5029735; doi:10.18632/oncotarget.8267)
Supplement: Supplementary file 1 [file oncotarget-07-24700-s001.pdf]

SUPPLEMENTARY FIGURES

A

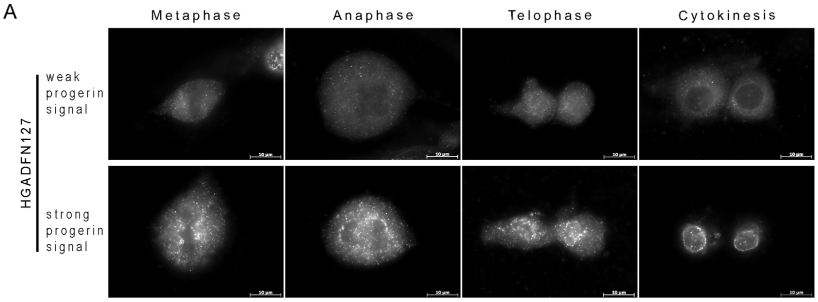

B

Lamin A localization %	Total Mitotic cells	Mitotic stages			
		Metaphase	Anaphase	Telophase	Cytokinesis
Progerin weak signal	49.79 ± 2.79	45.05 ± 1.29	48.21 ± 0.81	51.11 ± 1.51	52.13 ± 1.67
out of which Lamin A is mislocalized	25.16 ± 2.67	30.49 ± 0.25	24.69 ± 0.22	23.91 ± 0.32	23.18 ± 0.20
Progerin strong signal	50.21 ± 2.79	54.95 ± 2.26	51.79 ± 1.11	48.89 ± 2.37	47.87 ± 2.23
out of which Lamin A is mislocalized	96.09 ± 0.73	95.00 ± 0.96	96.55 ± 0.39	96.97 ± 0.91	96.73 ± 0.73

C

Lamin B localization %	Total Mitotic cells	Mitotic stages			
		Metaphase	Anaphase	Telophase	Cytokinesis
Progerin weak signal	50.20 ± 0.58	50.18 ± 1.56	50.00 ± 0.92	49.34 ± 1.81	51.27 ± 1.68
out of which Lamin B is mislocalized	18.47 ± 2.36	20.86 ± 0.31	20.50 ± 0.21	19.64 ± 0.31	18.52 ± 0.28
Progerin strong signal	49.80 ± 0.58	49.82± 2.19	50.00 ± 1.16	50.66 ± 2.28	48.73 ± 2.18
out of which Lamin B is mislocalized	97.65 ± 0.64	97.10 ± 0.67	98.00 ± 0.41	98.26 ± 0.76	97.40 ± 0.63

D

Emerin localization %	Total Mitotic cells	Mitotic stages			
		Metaphase	Anaphase	Telophase	Cytokinesis
Progerin weak signal	50.15 ± 1.34	48.26 ± 1.45	49.18 ± 0.86	51.26 ± 1.91	49.48 ± 1.73
out of which Emerin is mislocalized	24.68 ± 4.43	31.96 ± 0.42	23.76 ± 0.20	19.67 ± 0.27	21.67 ± 0.49
Progerin strong signal	49.85 ± 1.34	51.74 ± 2.22	50.82 ± 1.18	48.74 ± 2.37	50.52 ± 2.17
out of which Emerin is mislocalized	97.14 ± 0.55	97.12 ± 0.75	97.86 ± 0.45	97.41 ± 0.89	96.73 ± 0.67

E

SUN 1 localization %	Total Mitotic cells	Mitotic stages			
		Metaphase	Anaphase	Telophase	Cytokinesis
Progerin weak signal	51.25 ± 1.48	49.83± 1.56	51.39 ± 0.76	51.17 ± 1.68	49.60± 1.31
out of which SUN1 is mislocalized	26.84 ± 1.72	29.66 ± 0.37	26.56 ± 0.14	25.89 ± 0.25	25.97 ± 0.28
Progerin strong signal	48.75 ± 1.48	50.17 ± 2.28	48.61 ± 1.20	48.83 ± 2.44	50.40 ± 2.31
out of which SUN1 is mislocalized	98.04 ± 0.81	98.63 ± 0.73	98.25 ± 0.45	98.94 ± 0.92	96.49 ± 0.89

F

NPC 414 localization %	Total Mitotic cells	Mitotic stages			
		Metaphase	Anaphase	Telophase	Cytokinesis
Progerin weak signal	49.74 ± 1.22	48.74 ± 2.05	50.57 ± 1.05	50.51 ± 2.04	51.52 ± 1.89
out of which NPC 414 is mislocalized	8.82 ± 0.76	9.28 ± 0.24	7.95 ± 0.13	9.33 ± 0.19	9.95 ± 0.19
Progerin strong signal	50.26 ± 1.22	51.26 ± 2.30	49.43 ± 1.17	49.49 ± 2.27	48.48 ± 2.02
out of which NPC 414 is mislocalized	97.20 ± 0.74	97.06 ± 0.86	97.67 ± 0.43	97.96 ± 0.74	95.67 ± 0.45

G

Aurora B localization %	Total Mitotic cells	Mitotic stages			
		Metaphase	Anaphase	Telophase	Cytokinesis
Progerin weak signal	50.85 ± 2.17	50.18 ± 1.95	52.59 ± 1.16	53.89 ± 2.26	51.77 ± 1.80
out of which Aurora B is mislocalized	11.85 ± 3.08	14.71 ± 0.17	12.02 ± 0.18	12.50 ± 0.26	11.71 ± 0.15
Progerin strong signal	49.15 ± 2.17	49.82 ± 2.18	47.41 ± 1.19	46.11 ± 2.38	48.23 ± 2.06
out of which Aurora B is mislocalized	95.33 ± 2.22	91.85 ± 0.90	95.15 ± 0.48	97.75 ± 0.84	96.86 ± 0.43

H

Cenp-F localization %	Total Mitotic cells	Mitotic stages			
		Metaphase	Anaphase	Telophase	Cytokinesis
Progerin weak signal	51.66 ± 1.92	53.75 ± 1.55	51.49 ± 0.92	48.84 ± 1.98	49.46 ± 1.71
out of which Cenp-F is mislocalized	17.30 ± 3.13	22.09 ± 0.57	19.23 ± 0.15	18.25 ± 0.31	17.49 ± 0.25
Progerin strong signal	48.34 ± 1.92	46.25 ± 2.29	48.51 ± 1.26	51.16 ± 2.25	50.54 ± 2.37
out of which Cenp-F is mislocalized	96.89 ± 1.49	97.30 ± 0.74	97.45 ± 0.53	97.73 ± 0.76	93.58 ± 1.08

Supplementary Figure S1: Evaluation of the frequency of mislocalized nuclear proteins in HGPS mitotic cells at different stages.

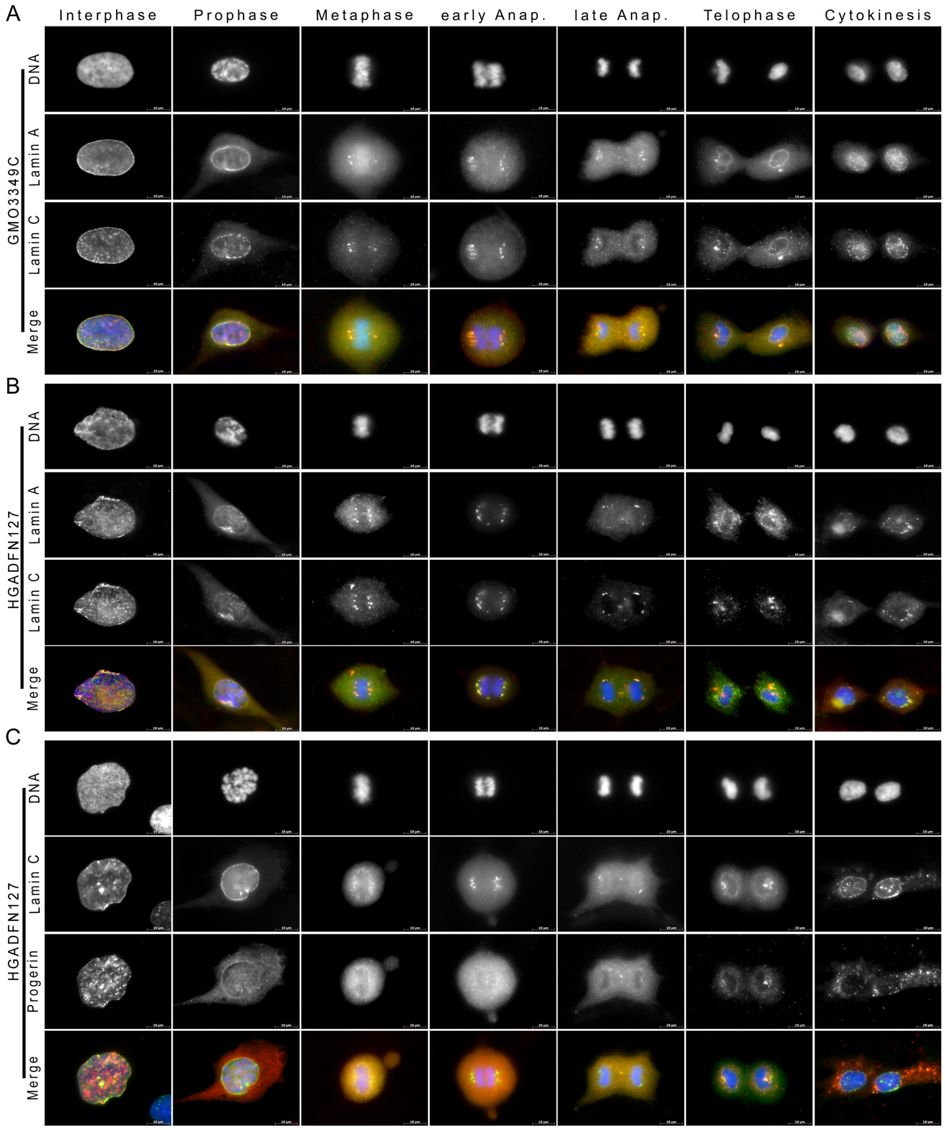

Supplementary Figure S2: Lamin A and Lamin C are colocalized during mitosis but their recruitment to daughter nuclei is delayed in HGPS cells.

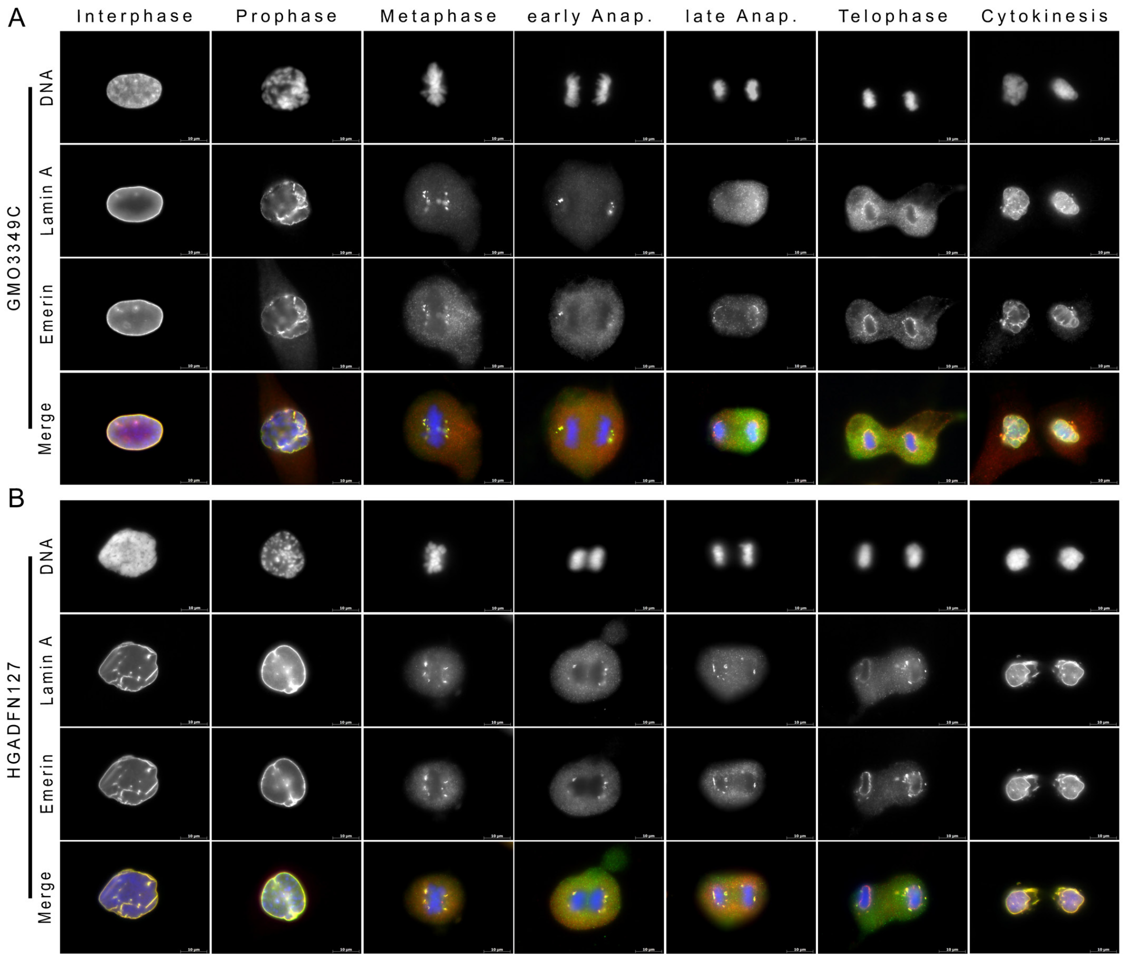

Supplementary Figure S3: Lamin A colocalized with Emerin but their recruitment to daughter nuclei is delayed in HGPS cells.

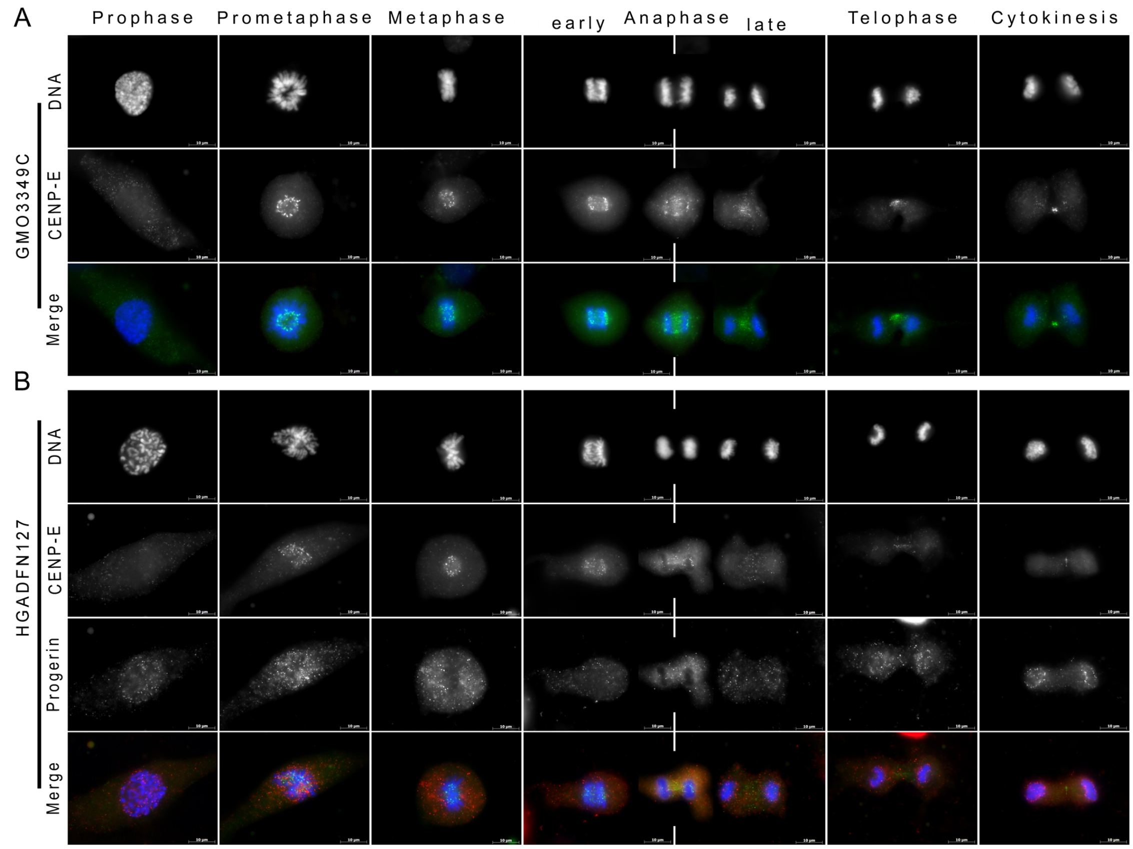

Supplementary Figure S4: CENP-E localization to the spindle midzone during anaphase is delayed in HGPS fibroblasts.

A

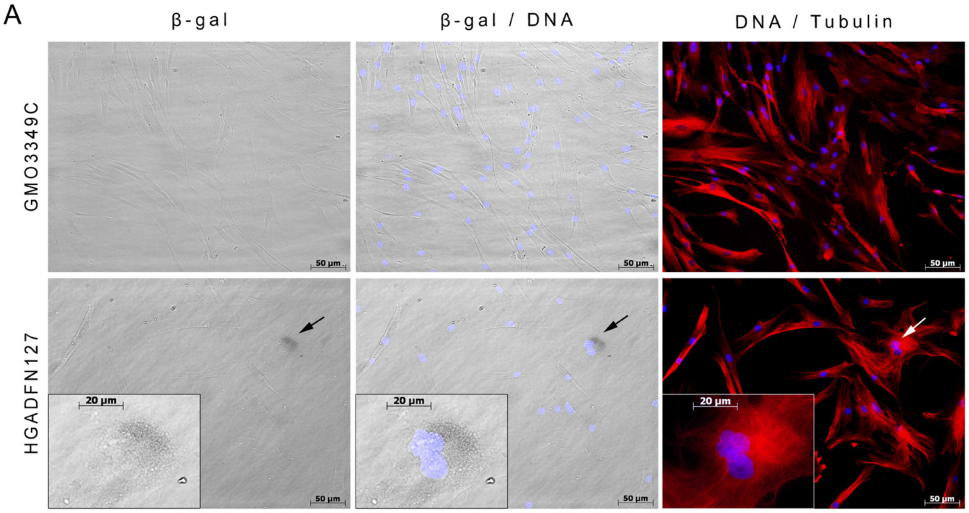

B

fibroblasts p10-p16	% β-gal positive	% bi-nucleated	% β-gal positive and binucleated
Control	1.34 ± 0.31	2.52 ± 0.74	0.56 ± 0.19
HGPS	6.42 ± 1.08	12.69 ± 1.68	2.88 ± 0.82

Supplementary Figure S5: Senescence detection using β-galactosidase staining revealed an increased number of senescent binucleated cells in the HGPS cultures.
